# S02-1 Physical activity and sedentary behaviour of children aged 6-9 in Europe: an analysis within the Childhood Obesity Surveillance Initiative (COSI)

**DOI:** 10.1093/eurpub/ckac093.006

**Published:** 2022-08-29

**Authors:** Stephen Whiting, Marta Buoncristiano, Peter Gelius, Karim Abu-Omar, Mary Pattison, Jolanda Hyska, Vesselka Duleva, Sanja Musić Milanović, Hana Zamrazilová, Tatjana Hejgaard, Mette Rasmussen, Eha Nurk, Lela Shengelia, Cecily C Kelleher, Mirjam M Heinen, Angela Spinelli, Paola Nardone, Akbota Abildina, Shynar Abdrakhmanova, Gulmira Aitmurzaeva, Zhamyila Usuopva, Iveta Pudule, Aušra Petrauskiene, Victoria Farrugia Sant’ Angelo, Enisa Kujundzic, Stevo Popovic, Anne-Siri Fismen, Ingunn Holden Bergh, Anna Fijalkowska, Ana Isabel Rito, Alexandra Cucu, Lacramioara Aurelia Brinduse, Valentina Peterkova, Andrea Gualtieri, Marta García-Solano, Enrique Gutiérrez-González, Zulfinissio Abdurrahmonova, Khadichamo Boymatova, Nazan Yardim, Maya Tanrygulyyeva, Daniel Weghuber, Karin Schindler, Dragana Stojisavljević, Aida Filipović Hadžiomeragić, Eliza Markidou Ionnaidu, Wolfgang Ahrens, Maria Hassapidou, Viktoria Anna Kovacs, Sergej M Ostojic, Lubica Ticha, Gregor Starc, Kenisha Russell Jonsson, Igor Spiroski, Harry Rutter, Romeu Mendes, Julianne Williams, Ivo Rakovac, João Breda

**Affiliations:** 1 Division of Country Health Programmes, WHO Regional Office for Europe, Copenhagen, Denmark; 2 EPIUnit – Instituto de Saúde Pública, Universidade do Porto, Porto, Portugal; 3 Department of Sport Science and Sport, FAU, Erlangen, Germany; 4 Nutrition and Food Safety Sector, Institute of Public Health, Tirana, Albania; 5 National Center of Public Health and Analyses, Sofia, Bulgaria; 6 School of Medicine, University of Zagreb, Croatian Institute of Public Health, Zagreb, Croatia; 7 Institute of Endocrinology, Obesity Management Centre, Prague, Czechia; 8 Danish Health Authority, Copenhagen, Denmark; 9 National Institute of Public Health, University of Southern Denmark, Copenhagen, Denmark; 10 National Institute for Health Development, Tallinn, Estonia; 11 National Center for Disease Control and Public Health, Tbilisi, Georgia; 12 National Nutrition Surveillance Centre, University College Dublin, Dublin, Ireland; 13 Italian National Institute of Health, Rome, Italy; 14 National Centre of Public Health of the Ministry of Health of the Republic of Kazakhstan, Nur-Sultan, Kazakhstan; 15 Republican Centre for Health Promotion, Bishkek, Kyrgyzstan; 16 Ministry of Health, Centre for Disease Prevention and Control, Riga, Latvia; 17 Lithuanian University of Health Sciences, Health Research Institute and Department of Preventive Medicine, Kaunas, Lithuania; 18 Primary Health Care, Floriana, Malta; 19 Institute of Public Health of Montenegro, Podgorica, Montenegro; 20 Faculty for Sport and Physical Education, University of Montenegro, Niksic, Montenegro; 21 Department of Health Promotion, Norwegian Institute of Public Health, Bergen, Norway; 22 Department of Health and Inequality, Division of Mental and Physical Health, Norwegian Institute of Public Health, Bergen, Norway; 23 Department of Cardiology, Institute of Mother and Child, Warsaw, Poland; 24 National Institute of Health Dr Ricardo Jorge I.P., Lisbon, Portugal; 25 National Institute of Public Health, Bucharest, Romania; 26 University of Medicine and Pharmacy Carol Davila, Bucharest, Romania; 27 The Endocrine Research Centre, Moscow, Russian Federation; 28 Health Authority San Marino, San Marino,Republic of San Marino; 29 Spanish Agency for Food Safety and Nutrition, Ministry of Health, Madrid, Spain; 30 Republican Centre for Nutrition, Ministry of Health and Social Protection of Population, Duschanbe, Tajikistan; 31 WHO Tajikistan Country Office, Dushanbe, Duschanbe, Tajikistan; 32 Diabetes and Metabolic Disorders Department, Ministry of Health, Public Health Institution, Ankara, Turkey; 33 Scientific Research Institute of Maternal and Child Health, Ashgabat, Turkmenistan; 34 Department of Pediatrics, Paracelsus Medical University, Salzburg, Austria; 35 Medical University of Vienna, Vienna, Austria; 36 Public Health Institute of Republic of Srpska, the University of Banja Luka, Faculty of Medicine, Banja Luka,Bosnia and Herzegovina; 37 Institute of Public Health of Federation of Bosnia and Herzegovina, Banja Luka,Bosnia and Herzegovina; 38 Ministry of Health, Limassol, Cyprus; 39 Leibniz Institute for Prevention Research and Epidemiology – BIPS, Bremen, Institute of Statistics, University of Bremen, Bremen, Germany; 40 International Hellenic University, Thessaloniki, Greece; 41 Hungarian School Sport Federation, Budapest, Hungary; 42 Faculty of Sport and PE, University of Novi Sad, Novi Sad, Serbia; 43 National Institute of Children Diseases, Medical Faculty of Comenius University, Bratislava, Slovakia; 44 Faculty of Sport, University of Ljubljana, Ljubljana, Slovenia; 45 Department of Living Conditions and Lifestyle, Public Health Agency of Sweden, Solna, Sweden; 46 Department of Physiology and Monitoring of Nutrition, Institute of Public Health, Skopje, Republic of Macedonia; 47 Department of Social and Policy Sciences, University of Bath, Bath, UK

**Keywords:** Screening time, screen-based activities, sleeping behaviour, family record form, parental questionnaire

## Abstract

**Background:**

Children are becoming less physically active for a variety of interrelated reasons. The availability of opportunities for safe active playgrounds, recreational activities and active transport has decreased, while time spend on sedentary screen-based activities has increased. This study aimed to evaluate physical activity (PA), sedentary and sleep behaviours of children aged 6-9 years in Europe using data from the WHO Childhood Obesity Surveillance Initiative (COSI).

**Methods:**

The fourth COSI data collection round was conducted in 36 countries from 2015-2018 using a standardized protocol including a family form completed by parents with specific questions about diet and physical activity-related behaviours.

**Results:**

Nationally representative data from the 24 countries, who filled in the non-mandatory family record form, were included. Information on PA, screen-time and sleep behaviours of 137,807 children were analysed. Pooled analysis showed that: one in two children walked or cycled to school every day; one in two children were members of a sport or dancing club; around 40 % of children spent at least two hours per day watching TV or using electronic devices; around four in five children were actively or vigorously playing each day; around 88 % of children slept for at least nine hours per night. Country specific analyses showed pronounced differences in prevalence estimates between countries.

**Conclusions:**

While the severity of the problem varies between countries, physical inactivity and sedentary behaviours are common across the European Region. Policy makers across the Region must do more in order to increase opportunities for young people to participate in daily activities. Furthermore, they should explore solutions to reduce the amount of time spend on sedentary activities, in order to halt the rise in overweight and obesity.

